# Response of the photosynthetic characteristics and antioxidant system of *Suaeda salsa* to the changes of underground brine depth

**DOI:** 10.3389/fpls.2024.1471742

**Published:** 2024-10-15

**Authors:** Ping Wang, Wenjing Xu, Zehao Zhang, Zhanyong Fu, Tian Li, Jingkuan Sun

**Affiliations:** Shandong Key Laboratory of Eco-Environmental Science for Yellow River Delta, Shandong University of Aeronautics, Binzhou, China

**Keywords:** underground brine depth, shell island, photosynthetic characteristics, antioxidant system, *Suaeda salsa*

## Abstract

**Introduction:**

Water and salt conditions are key factors influencing vegetation growth on shell island in the Yellow River Delta. However, the effects of the depth of underground brine on the photosynthetic characteristics and antioxidant system of halophytes remain unclear.

**Methods:**

The laboratory simulation experiment was carried out to investigate the effect of the changes of underground brine depth on *Suaeda salsa* using four levels of groundwater: 0 cm, 15 cm, 30 cm and 45 cm.

**Results:**

The results showed that different underground brine depths had significant impacts on the photosynthetic characteristics and antioxidant system of *S. salsa*, and 0-30 cm underground brine depth was suitable for *S. salsa* growth. The net photosynthetic rate (Pn), transpiration rate (Tr), stomatal conductance (Gs), light utilization efficiency (LUE) and carboxylation efficiency (CE) of *S. salsa* increased first and then decreased with increasing depth of underground brine. The stomatal limitation value (Ls) and WUE of *S. salsa* reached the peak value at the groundwater depth of 0 cm, and water use efficiency was reduced by 19.4%, 8.0% and 8.6% at 15 cm, 30 cm, and 45 cm, respectively, compared to the 0 cm treatment. With the deepening of underground brine depth, the value of LUE and CE firstly increased and then decreased, and reached the peak value when the depth was 30 cm. The antioxidant enzyme (SOD, POD and CAT) activities of *S. salsa* decreased and then increased with the increase of underground brine depth. The enzyme activities were the lowest when the underground brine depth was 30 cm. As the groundwater depth increased, MDA content decreased and then increased. The highest degree of membrane peroxidation in *S. salsa* leaves was observed at the depth of 45 cm.

**Discussion:**

Our study reveals that the antioxidant capacity of *S. salsa* was weakened at the underground brine depth of 45 cm and the growth of *S. salsa* was inhibited.

## 1 Introduction

The shell island of the Yellow River Delta is the most typical coastal estuarine wetland in the world, with unique ecosystems and important ecological functions, which plays an essential role in materials production and biodiversity conservation (Zhang et al., 2023). In recent years, natural and anthropogenic disturbances, such as agricultural cultivation, coastal erosion, and oil pollution, have increased complexity of water and salt transport processes and seriously impaired the health and function of coastal wetland ecosystems (Li et al., 2021; Wang et al., 2022; Xia et al., 2019).

Due to the simultaneous interaction of the river, sea and land, and the superimposed influence of human activities, the groundwater dynamics of the Yellow River Delta are very complex (Hou et al., 2022; Lv et al., 2017). Underground brine depth is a critical factor affecting vegetation growth and development in the shell island ecosystem (Guan et al., 2012; Ren et al., 2019). Plants have evolved various ways to adapt to different underground brine depths during their long-term growth and development, thus allowing them to survive and reproduce under stressful conditions. Previous studies showed that the mechanism of groundwater affecting the photosynthesis of plants is complex (Xia et al., 2017). When plants are stressed by water, their physiological processes and internal structures will change, including the closure of stomata and the reduction of enzyme activities related to photosynthetic processes, which eventually cause a lower rate of photosynthetic (Chen et al., 2011; Law and Finch, 2011; Liu et al., 2022). Therefore, when considering the photosynthetic characteristics of plants, a series of physiological and biochemical indicators should be taken into consideration, including antioxidant enzyme activities, during photosynthesis, to have a more accurate understanding of the photosynthetic characteristics response of *S. salsa* on underground brine depth (Zhang et al., 2011). SOD, POD, and CAT can scavenge excess reactive oxygen of plants in water-stressed adversity and increase plant resistance (Dong et al., 2013). Plant organs also undergo membrane lipid peroxidation under adversity, which leads to the accumulation of MDA (Dong et al., 2013; Maevskaya and Nikolaeva, 2013). The underground brine depth serves as an important factor limiting plant recovery in the shell island, and *S. salsa* growth is severely inhibited by frequent inundation due to seawater intrusion. Therefore, the study of photosynthetic characteristics and antioxidant enzyme activities of plants under water and salt stress is more conducive to the in-depth understanding of the physiological and ecological regulatory mechanisms of plants, which is of great significance in revealing the salt-tolerant characteristics of plants and improving the ecological environment of the region.

The vegetation types on shell island are mainly dominated by salt-tolerant shrubs and herbs, such as *Tamarix chinensis*, *Periploca sepium*, *Astragalus adsurgens*, and *Suaeda salsa* (Chen et al., 2019). Among them, *S. salsa* is the main primary producer and a typical dominant herb in the coastal zone of the Yellow River Delta, with multiple functions such as sand fixation and coastal erosion resistance, which are essential for maintaining the ecosystem stability (Dong et al., 2024). Previous studies about *S. salsa* have primarily concentrated on the influence for water and salt stress as well as nitrogen and phosphorus addition on physiological characteristics, growth and development, stoichiometric relationships, and photosynthetic properties (Li et al., 2020; Ma et al., 2013). However, few studies have been conducted on the effects of the depth of underground brine on the photosynthetic characteristics and antioxidant system of *S. salsa*. The effects of underground brine stress on the physiological characteristics of *S. salsa* in the coastal area and the synergistic change pattern of their photosynthesis need to be further explored, which is of great significance in elucidating the adaptive strategies of coastal plants in response to changes in underground brine.

Therefore, the aim of this study is to increase the research on the effects of underground brine depth on the photosynthetic characteristics and antioxidant systems of *S. salsa* under water and salt stress conditions and to reveal the adaptive mechanism of plant photosynthetic characteristics and antioxidant system to underground brine depth. Additionally, this study try to answer the following questions: (1) What is the threshold of tolerance for *S. salsa* to the depth of underground brine water on the shell island? (2) What is the interaction relationship between plant photosynthetic properties and antioxidant enzyme activity parameters? (3) How does *S. salsa* adapt to changes in underground brine depth through plant regulation?

## 2 Materials and methods

### 2.1 Experimental materials

The *S. salsa* seeds and shell sand for the experiment were collected in shell island Binzhou City, Shandong Province (117°56′20″E, 38°14′05″N). The collected seeds were air-dried and sealed for storage at 4°C. Plastic pots (25 cm in diameter and 50 cm in height) with holes punched on the side and filled with shell sand were used for planting. Four plastic buckets were used to store brine. The experiments were conducted in a greenhouse of 25°C average temperature and 45% average relative humidity.

### 2.2 Experimental design

**Figure 1:**
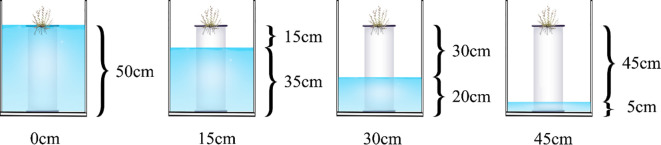
Experimental design.

In May 2020, 30 seeds were sown in each pot and sprinkled with a little water. Then, the plastic pots were moved separately to plastic buckets, and different depths of 0.8% brine were added (simulated groundwater salt concentration). According to the actual groundwater burial depth of the shell islands in the Yellow River Delta, four brine depth treatments were set: 50 cm, 35 cm, 20 cm, and 5 cm, respectively (**Figure 1**). Underground brine depths were 0 cm, 15 cm, 30 cm, and 45 cm, respectively. Set up three repetitions in each bucket. Seeds started to germinate after 3 days of sowing, and, then, 12 plantings were set in each pot after 14 days. The distilled water was added to the plastic bucket daily to compensate for the evaporation of water.

### 2.3 Photosynthetic characteristics determination

CID-340 photosynthesis system was applied to determine the net photosynthetic rate (Pn), transpiration rate (Tr), intercellular CO_2_ concentration (Ci), and stomatal conductance (Gs) in the third leaf below the apical growth point of the *S. salsa*. Photosynthetic characteristics were measured with the photosynthetically active radiation (PAR) of 1,000 μmol m^−2^ s^−1^ and a CO_2_ concentration of 390 μmol mol^−1^. Measurement time is about 40 days after germination. Stomatal limitation value (Ls), WUE, light utilization efficiency (LUE), and carboxylation efficiency (CE) were calculated by the formula (Chen et al., 2024; Zhong et al., 2019).


Ls=1-Ci/Ca

WUE=Pn/Tr

LUE=Pn/PAR

CE=Pn/Ci

### 2.4 Antioxidant enzyme activity and MDA determination

SOD, POD, CAT, and MDA were measured by the kit. The procedure for the determination was performed according to the kit instructions (Sun et al., 2019). Among them, SOD activity was measured using Nitrotetrazolium Blue chloride (NBT) method (Stewart and Bewley, 1980), POD activity was determined using guaiacol methods (Frébort et al., 1992), CAT activity was determined using ultraviolet absorption (Frébort et al., 1992), and MDA content was measured using thiobarbituric acid method (Xia et al., 2023).

### 2.5 Statistical analysis

One-way ANOVA and the Duncan test were used to compare the effects of underground brine depth on the photosynthetic characteristics and antioxidant system of *S. salsa*. The Pearson test was used for the correlation analysis.

## 3 Results

### 3.1 Effect of underground brine depth on photosynthetic characteristics

**Figure 2:**
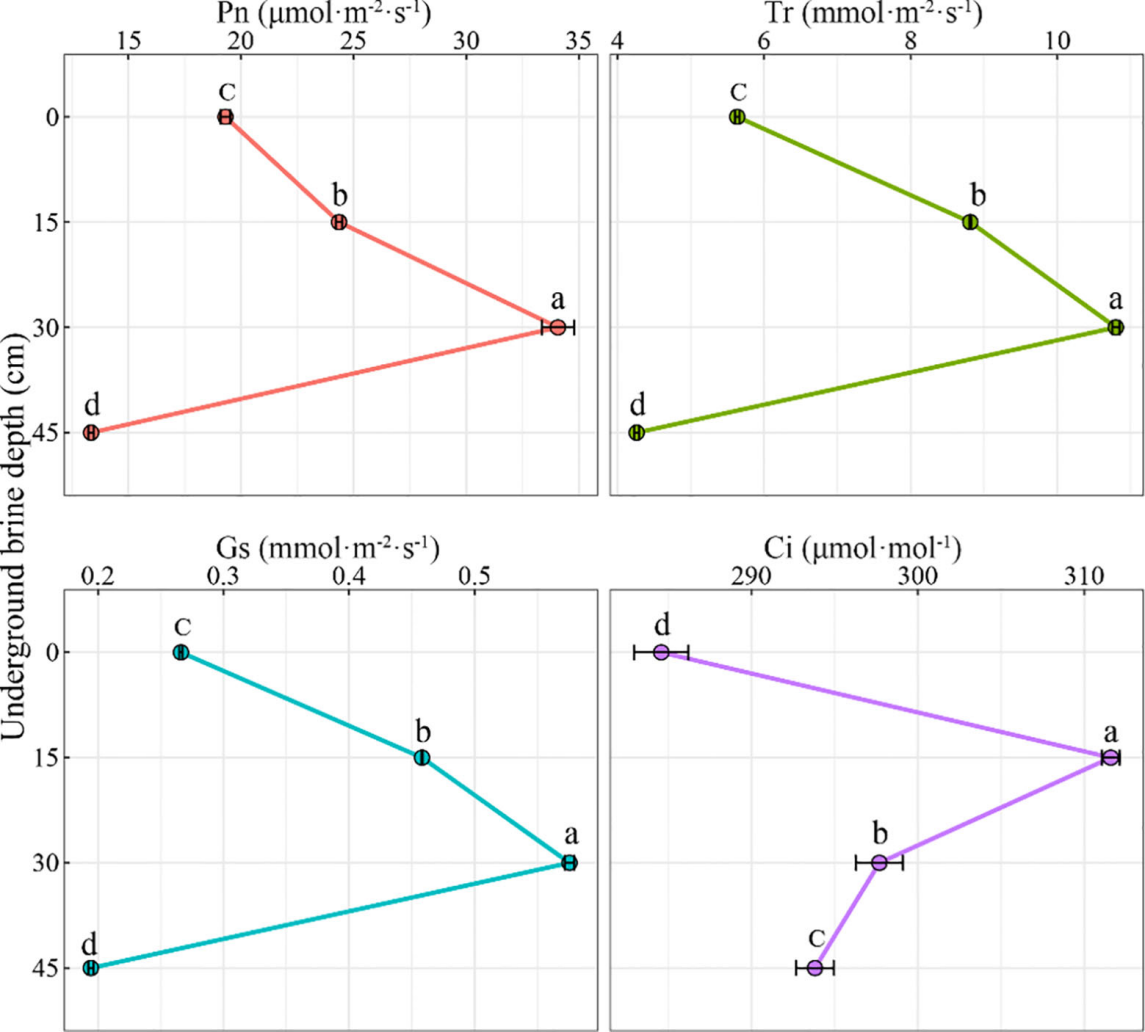
Effect of underground brine depth on photosynthetic characteristics of *S. salsa.* Different lowercase letters indicate that the difference among treatments is significant.

The effect of underground brine depth on the photosynthetic characteristics of *S. salsa* was showed in **Figure 2**. It was found that Pn tended to increase and then decrease with the increase of the underground brine depth. At 30 cm, Pn reached the maximum. At 30 cm, Pn reached the maximum. Compared to that at 0 cm, Pn at 15 cm and 30 cm significantly increased by 26.1% and 76.4% (*P* < 0.05), respectively. At 45 cm, Pn reached the minimum and was significantly reduced by 30.9% compared to that at 0 cm. The trend of Tr and Gs with underground brine depth was consistent with that of Pn. At 30 cm, Tr and Gs were remarkably higher than those at 0 cm (*P* < 0.05), whereas, at 45 cm, both reached the minimum and were remarkably lower than those at 0 cm (*P* < 0.05). At 15 cm, 30 cm, and 45 cm, Gs was remarkably higher than that at 0 cm (*P* < 0.05).

### 3.2 Effect of underground brine depth on resource utilization efficiency

**Figure 3:**
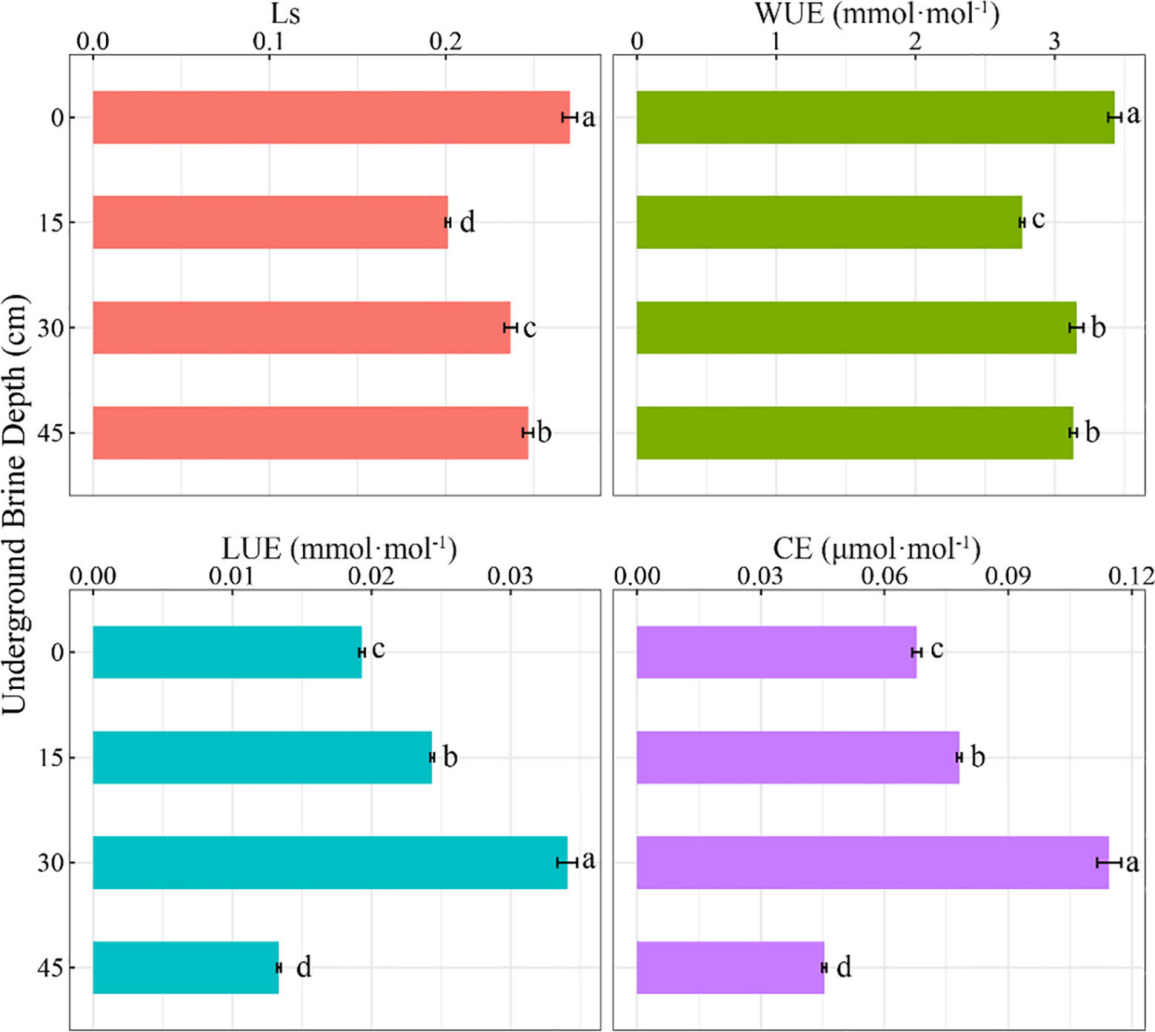
Effects of underground brine depth on resources utilization efficiency of *S. salsa*. Different lowercase letters indicate that the difference among treatments is significant.

Different underground brine depths affected the resource use efficiency of *S. salsa* significantly (**Figure 3**). Ls and WUE at 15 cm, 30 cm, and 45 cm treatments were significantly lower than that at 0 cm (*P* < 0.05). At 15 cm, 30 cm, and 45 cm, WUE was reduced by 19.4%, 8.0%, and 8.6%, respectively. As the depth of the underground brine increases, CE and LUE tend to first increase and then decrease. At 45 cm treatment, LUE and CE were significantly suppressed and decreased by 30.9% and 33.1%, respectively (*P* < 0.05). At 15 cm and 30 cm treatments, LUE and CE were significantly higher than those at 0 cm (*P* < 0.05).

### 3.3 Effect of underground brine depth on antioxidant system

**Figure 4:**
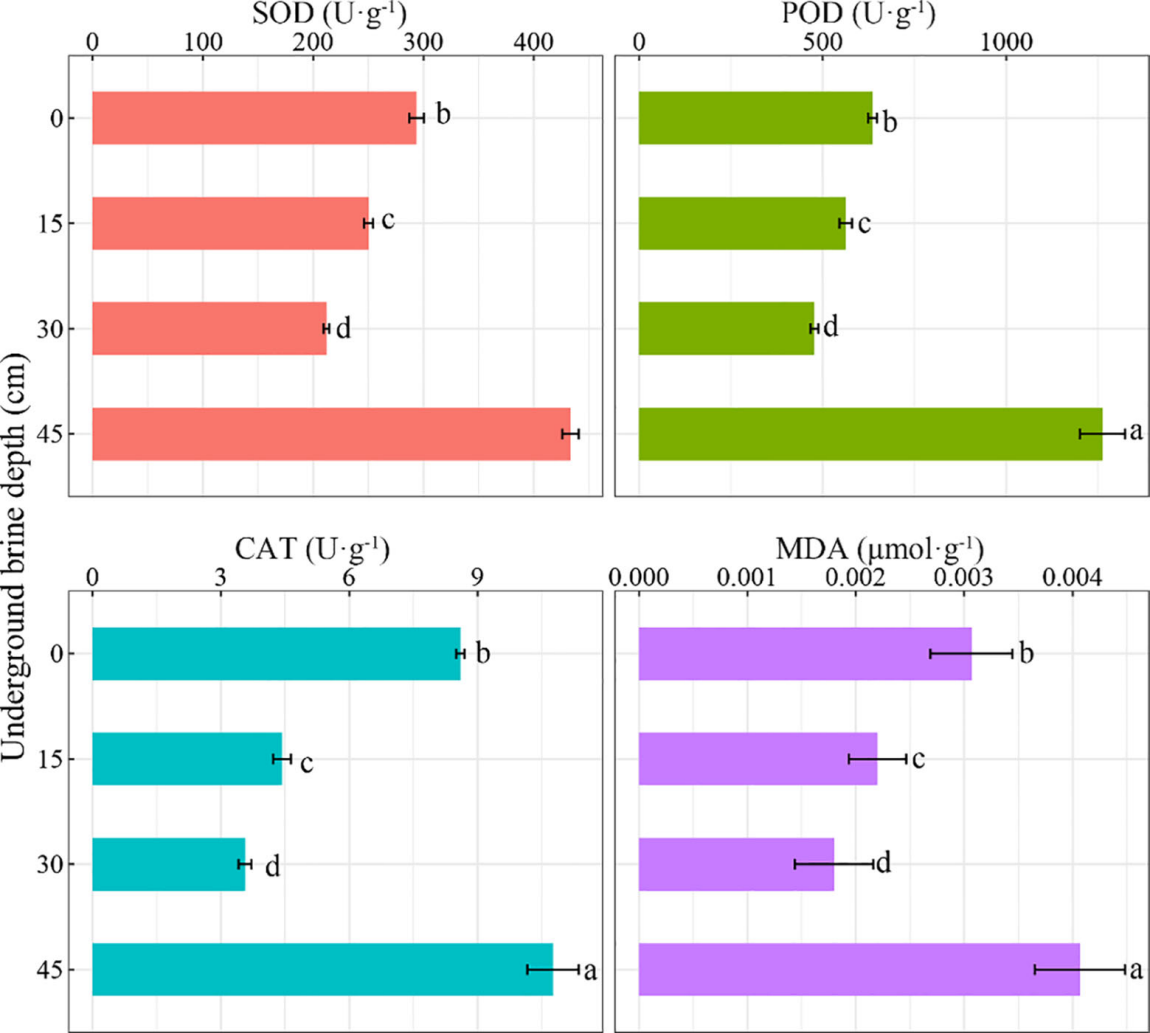
Effects of underground brine depth on antioxidant system of *S. salsa*. Different lowercase letters indicate that the difference among treatments is significant.

The SOD, POD, and CAT activities decreased and then enhanced with increasing underground brine water level and peaked at 45 cm treatment (**Figure 4**), and the activity of the three enzymes was significantly higher at 45 cm than that of other treatments (*P* < 0.05). The activity of the three enzymes at 30 cm treatment reached the minimum value and was significantly lower than that at 0 cm (*P* < 0.05). The MDA content followed the same trend as the three enzyme activities, with the lowest at 30 cm treatment and the highest at 45 cm treatment.

### 3.4 Correlation analysis between photosynthetic characteristics and antioxidant system

**Figure 5:**
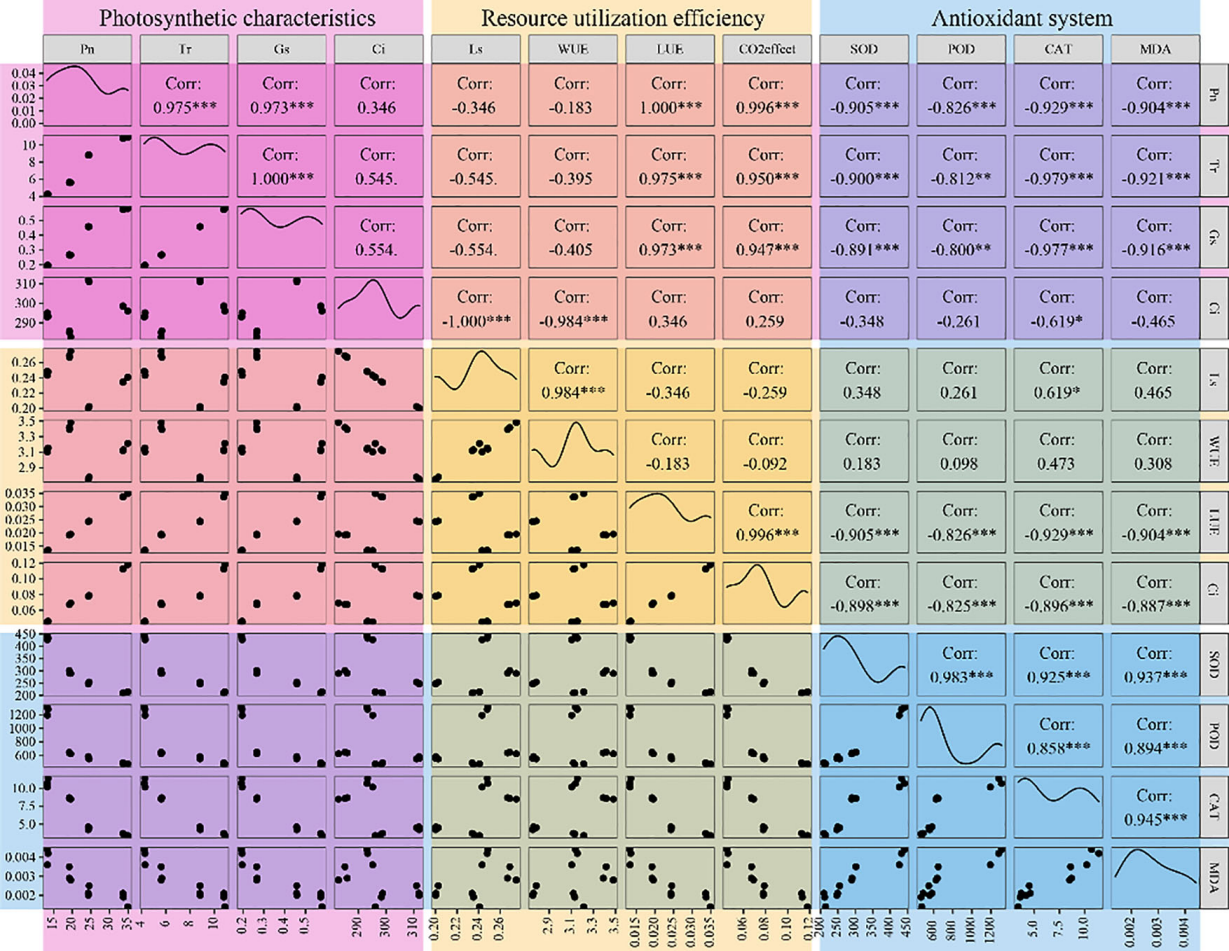
Correlation analysis of between photosynthetic characteristics and antioxidant system. *, **, and *** indicate that the correlation is significant at 0.05, 0.01, and 0.001 level, respectively.

There were significant correlations between the photosynthetic characteristics of *S. salsa* (**Figure 5**). Pn was significantly and positively correlated with Tr and Gs, respectively, and was significant at the 0.01 level, whereas there was no significant correlation between Ci and Pn, Tr, and Gs, respectively. There were significant positive correlations between MDA content, SOD, POD, and CAT activities (*P* < 0.01). The SOD, POD, and CAT activities and MDA content were negatively correlated with Pn, Tr, and Gs, respectively (*P* < 0.01). The negative correlation between Ci and CAT activity was found (*P* < 0.01).

**Figure 6:**
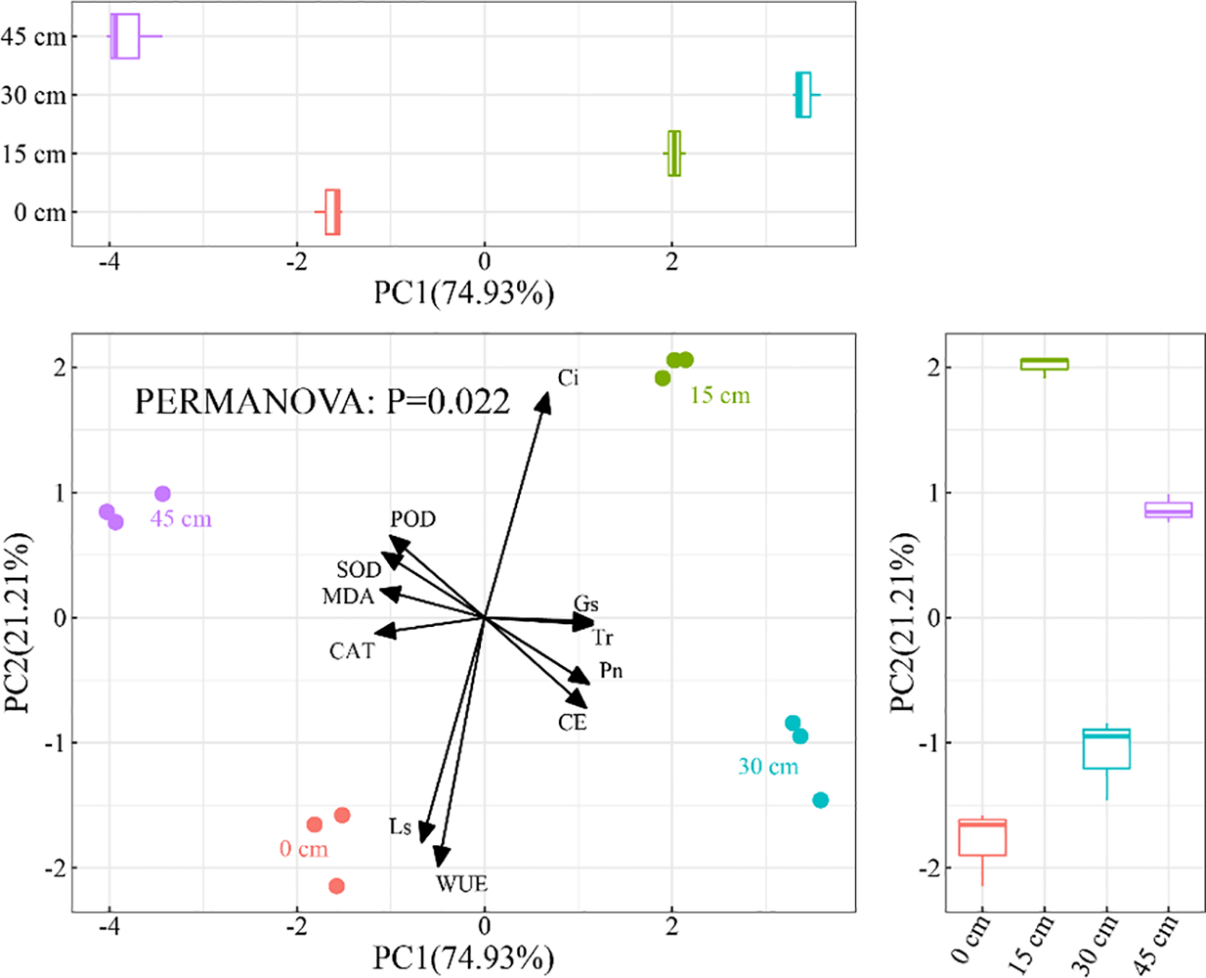
PCA of photosynthetic characteristics and antioxidant system.

The two axes of the principal component analysis (PCA) explained 74.93% and 21.21% of the variation in photosynthetic characteristics and antioxidant systems of *S. salsa*, respectively (**Figure 6**). PCA showed that the four underground brine depths of burial were located in different quadrants, indicating that there were large effects of underground brine depth on the photosynthetic characteristics and the antioxidant system of *S. salsa*. PERMANOVA further showed that the underground brine depth of burial explained 40.44% of the variation in photosynthetic characteristics and antioxidant systems of *S. salsa* (*P* < 0.05).

## 4 Discussion

The spatial distribution of groundwater level and shallow groundwater can influence the growth of natural vegetation in the region (Xu and Su, 2019). There are different tolerance thresholds and corresponding physiological adaptation strategies of various plants to the underground brine depth. Investigating the response mechanisms of plants to the underground brine depth is critical to the restoration of degraded ecosystems (Jin et al., 2014; Zhang et al., 2013). In this study, it was found that *S. salsa* could survive in the 0- to 45 cm range of underground brine depth. However, the plant was significantly suppressed at the 45 cm depth. The deeper underground brine depth may have caused a water deficit of *S. salsa*, resulting in their growth inhibition. The adaptation of different plants to the underground brine depth is different. An et al. (2011) found that *Tamarix chinensis* was mainly distributed in the supralittoral zone with deeper underground water depths, *Phragmites communis* was widely distributed in wetlands of estuaries and coastal marshes with moderate underground water depths, whereas *S. salsa* was distributed in coastal mudflats with shallow underground water depths (Xing and Xing, 2019). This finding is similar to our study. The shorter root system of *S. salsa* leads them to live in habitats with shallow water depths. In addition, the underground brine depth is directly related to whether the soil capillary water can reach the surface and determines the soil salt content (Dou et al., 2019). It has been found that, when the underground water depth is less than 0.5 m, the effect of underground water depth on soil salt content is larger (Ma et al., 2013). The change in groundwater level causes a change in soil salt content. This suggests that *S. salsa* is influenced by both the underground water depth and soil salt content. The fact that *S. salsa* can grow normally and maintain a high survival rate at different gradients of groundwater depth indicates that they are highly adaptable to changes in the water–salt environment of shallow groundwater (He et al., 2021), which is the main reason that *S. salsa* can become the dominant species on shell island.

For dominant plant species growing in tidelands, the ability to adapt to different gradients of underground brine depth is essential for their survival. As the dominant plant on the shell island in the Yellow River Delta, *S. salsa* has developed corresponding strategies in plant physiology and morphology to adapt to the changing groundwater level. To tolerate external stresses, plant leaves optimize gas exchange efficiency via regulating stomatal size, density, and opening (Xin et al., 2024). A study has shown that being under salt stress conditions can increase the stomatal density and decrease the stomatal opening of plant leaves (Zhang et al., 2018a). In this study, it was found that the underground brine depth changed the photosynthetic characteristics and resource use efficiency of *S. salsa*. The Pn, Tr, Gs, LUE, and CE of *S. salsa* increased with increasing depth when the underground water depth was between 0 cm and 30 cm. This indicated that the *S. salsa* can actively adapt to the changing underground brine depth by regulating its photosynthetic characteristics and resource utilization efficiency to ensure its normal growth. At the 45 cm depth, photosynthesis decreased, indicating that the growth of *S. salsa* was inhibited at this depth. The WUE peaked at 0 cm depth and decreased significantly at 15–45 cm. This result indicates that *S. salsa* has the highest water use efficiency when water is sufficient. The reduced water use efficiency of *S. salsa* at 15 to 45 cm depth may be related to water scarcity. Stomatal limitation theory suggested that the factors limiting plant photosynthesis are divided into stomatal and non-stomatal factors. The change of Ci is the main basis for judging stomatal restriction or non-stomatal restriction. When Pn, Gs, and Ci decreased, whereas Ls increased, it indicated that plant photosynthesis was mainly limited by stomata (Chen et al., 2024). Photosynthesis is the fundamental process that provides the material and energy necessary for plant growth and development. However, under salt stress, the photosynthetic efficiency of plant leaves is reduced (Zhang et al., 2018b; Wei et al., 2024). In this study, when the underground brine depth varied from 30 cm to 45 cm, Pn decreased rapidly, and Ci decreased with the decrease of Gs, whereas Ls increased, indicating that the decrease of Pn was caused by stomatal factors (Li et al., 2012).

Shallow groundwater is usually considered to be the major water source for plant growth in the Yellow River Delta region (An et al., 2013). Recent research on the impact of underground brine depth mainly focused on plant physiological characteristics, functional traits, and changes in photosynthetic properties (Wei et al., 2020). The response of plant antioxidant system to underground brine depth was less studied. A previous study has indicated that plants usually increase stress tolerance via adjusting their SOD, POD, and CAT enzyme activities when they are stressed (Li et al., 2013). Plant leaf protective enzyme systems are damaged under salt stress, and plant oxidase activity is reduced (Wei et al., 2024). Liu and Zhong (2016) discovered that *Leymus chinensis* enhanced salinity tolerance via enhancing antioxidant enzymes activity. *S. salsa* is subjected to gradually intensified drought stress with the deepening of the underground brine level, thus enhancing its stress resistance by increasing its antioxidant enzyme activities. Li et al. (2010) showed that the inhibitory effects of POD and CAT activities on MDA content in *Periploca sepium* leaves were insignificant under drought stress, whereas the inhibitory effects of SOD activity on MDA content were significant. In this study, the inhibitory effect of the increased activities of the three antioxidant enzymes on the MDA content in the *S. salsa* plants was insignificant. Guan et al. (2011) found that the CAT activity of *S. salsa* peaked at 30 cm underground water depth by water–salt stress experiment, which is consistent with our result. Differently, Guan et al. (2011) discovered that *S. salsa*’s MDA content was higher at 0-cm underground water depth. This may be attributed to differences in cultivation substrates. The weak water-holding capacity of shell sand and the easy evaporation of water lead to an increase in soil salt (Chen et al., 2022). These reasons may have aggravated the drought stress and salt stress of *S. salsa* and increased the MDA content (Shailani et al., 2021). Increased drought stress leads to an increased membrane lipid peroxidation exceeding the antioxidant capacity of the plant. This indicates that the resistance of *S. salsa* is weakened when the underground brine depth is deeper. At the same time, the photosynthetic properties of *S. salsa* were also significantly inhibited. Therefore, the underground brine depth should be considered in vegetation restoration on shell islands.

## 5 Conclusion

*S. salsa* can grow normally in the underground brine depth range of 0 cm to 45 cm and adapt to the changing stress of brine depth by regulating its photosynthetic and antioxidant systems. *S. salsa* had the greatest Pn and the lowest antioxidant enzyme activity at 30 cm underground brine depth. SOD, POD, and CAT activities and MDA content were negatively correlated with Pn, Tr, and Gs, respectively. At 45 cm underground brine depth, the photosynthetic properties of *S. salsa* were inhibited, antioxidant capacity was reduced, MDA content was highest, and growth was significantly inhibited. When the underground brine depth was 45 cm, the growth of *S. salsa* was subjected to drought stress, which stimulated an increase in antioxidant enzyme activity. The growth of *S. salsa* in shell sands is more susceptible to the underground brine depth. The underground brine depth must be taken into consideration when conducting ecological restoration work on shell island.

## Data Availability

The original contributions presented in the study are included in the article/supplementary material. Further inquiries can be directed to the corresponding author.
